# Individual Cryptic Scaling Relationships and the Evolution of Animal Form

**DOI:** 10.1093/icb/icz135

**Published:** 2019-07-31

**Authors:** W Anthony Frankino, Eric Bakota, Ian Dworkin, Gerald S Wilkinson, Jason B Wolf, Alexander W Shingleton

**Affiliations:** 1 Department of Biology and Biochemistry, University of Houston, Houston, TX 77204-5001, USA; 2 Department of Biology, McMaster University, Hamilton, Ontario, Canada L9H 6X9; 3 Department of Biology, University of Maryland, College Park, MD 20742, USA; 4 Milner Centre for Evolution and Department of Biology and Biochemistry, University of Bath, Bath BA2 7AY, UK; 5 Department of Biological Sciences, University of Illinois at Chicago, Chicago, IL 60607, USA

## Abstract

Artificial selection offers a powerful tool for the exploration of how selection and development shape the evolution of morphological scaling relationships. An emerging approach models the expression and evolution of morphological scaling relationships as a function of variation among individuals in the developmental mechanisms that regulate trait growth. These models posit the existence of genotype-specific morphological scaling relationships that are unseen or “cryptic.” Within-population allelic variation at growth-regulating loci determines how these individual cryptic scaling relationships are distributed, and exposure to environmental factors that affect growth determines the size phenotype expressed by each individual on their cryptic, genotype-specific scaling relationship. These models reveal that evolution of the intercept and slope of the *population*-level static allometry is determined, often in counterintuitive ways, largely by the shape of the distribution of these underlying *individual*-level scaling relationships. Here we review this modeling framework and present the wing-body size individual cryptic scaling relationships from a population of *Drosophila melanogaster*. To determine how these models might inform interpretation of published work on scaling relationship evolution, we review studies where artificial selection was applied to alter the parameters of population-level static allometries. Finally, motivated by our review, we outline areas in need of empirical work and describe a research program to address these topics; the approach includes describing the distribution of individual cryptic scaling relationships across populations and environments, empirical testing of the model’s predictions, and determining the effects of environmental heterogeneity on realized trait distributions and how this affects allometry evolution.

## Introduction

It is suggested that selection provides a valuable tool for studying the constancy or labiality of the growth patterns which determine morphology—(Robertson 1962)

The evolution of morphological scaling relationships is a primary mechanism by which morphological diversification occurs. Traits that evolve steep scaling relationships represent striking examples of exaggerated ornaments, weapons, and specialized tools (e.g., Huxley 1932; Gould 1974; Wilkinson and Dodson 1997; Larabee et al. 2017). Traits that evolve shallow scaling relationships are perhaps less conspicuous (e.g., Koh et al. 2005; Eberhard 2009) but are equally interesting developmentally, ecologically, and evolutionarily. Despite intense interest in how scaling relationships are maintained within biological groups (e.g., sexes, populations, species, etc.), and how they are modified to generate morphological diversity among groups, surprisingly little is known conclusively regarding the role of selection in these processes.

Humans have used artificial selection to create domestic breeds with desirable proportions for millennia (Clutton-Brock 1992; Larson and Fuller 2014). However, it is only 70 years since Robertson and Reeve’s (1952) pioneering work (Robertson 1962) established artificial selection as a way to investigate the evolutionary malleability of relative trait size, body proportion, and morphological scaling. Since then, several studies have used artificial selection to determine the evolutionary independence of the intercept and slope of the allometric equation (e.g., Egset et al. 2012; Bolstad et al. 2015; Stillwell et al. 2016); the relative roles of natural selection and developmental constraint in scaling relationship expression and evolution (e.g., Frankino et al. 2005, 2007; Kotrschal et al. 2013; Bolstad et al. 2015; Booksmythe et al. 2016); the heritability of individual traits or the scaling relationship between them (e.g., Weber 1990; Wilkinson 1993; Emlen 1996); the role of sexual selection in the production of relative trait size (e.g., Ewing 1961; Wilkinson and Reillo 1994; Cayetano et al. 2011; Menezes et al. 2013; Booksmythe et al. 2016), etc. (See reviews in Baker and Wilkinson 2001; Frankino et al. 2009; Shingleton and Tang 2012; Pélabon et al. 2014; Voje et al. 2014; Houle et al. 2019.)

**Fig. 1:**
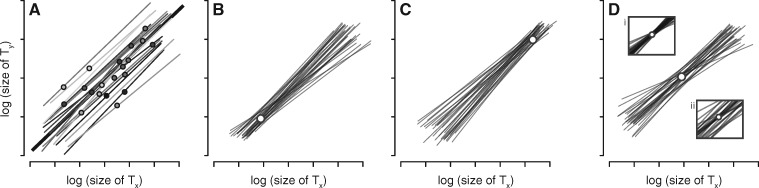
Relationship among individual cryptic scaling relationships, the population-level static allometry, and the mean point of intersection. (**A**) Individual cryptic scaling relationships (thin lines) between two traits (*t*_1_ and *t*_2_; e.g., wing and body size, respectively) for a set of genotypes. Variation in an environmental factor that affects growth (e.g., access to nutrition) determines which size phenotype (round markers) each genotype expresses on its individual cryptic scaling relationship. The population-level static allometry (thick line) is fit using all expressed phenotypes in the population. (**B–D**) Individual cryptic scaling relationships can have three distributions, depending on the location of their MPI (open circles) relative to the potentially expressed size phenotypes of individuals in the population: (**B**) If the MPI is at the lower end of the size range, the pattern is referred to as a “speedometer,” (**C**) if the MPI is in the higher end of the range of size, the pattern is called a “broomstick,” and (**D**) if it is near the center of the size range, the pattern is a “seesaw.” In reality, natural populations likely exist along the continuum represented by these three idealized distributions, and a given distribution might exhibit variation in intersection points around the MPI (insets in panel D).

In tandem with these empirical studies, a variety of mathematical treatments have sought to identify how natural selection might transform one pattern of scaling into another (e.g., Lande 1979; Bonduriansky and Day 2003; Kodric-Brown et al. 2006; Fromhage and Kokko 2014). New information regarding the developmental regulation and integration of trait growth has been used increasingly to study the expression and evolution of morphological scaling relationships; space limitations preclude a discussion here of the mechanisms underlying growth regulation, however, interested readers are directed to several recent reviews for details (e.g., Emlen and Allen 2003; Shingleton et al. 2007, 2008; Shingleton 2011; Shingleton and Frankino 2013, 2018; Warren et al. 2013; Nijhout et al. 2014; Mirth et al. 2016; Casasa and Moczek 2019; Cooper 2019). Some studies model formally the role of development in scaling expression (e.g., Nijhout and Wheeler 1996; Shingleton et al. 2008) and an emerging approach distinguishes between two types of scaling relationships: *individual-level* cryptic scaling relationships and the well-established concept of the *population-level* static allometry (Fig. 1; Dreyer et al. 2016; Houle et al. 2019; Shingleton 2019). Distinguishing between these classes of scaling relationship generates potentially revealing ways of thinking about the mathematical representation of morphological scaling relationships, the developmental mechanisms that selection targets to alter scaling, how genetic variation among individuals in these mechanisms influences the response to selection, and the importance of environmentally-induced size variation in the evolution of scaling (Shingleton et al. 2007, 2008; Dreyer et al. 2016; Shingleton 2019). Below, we sketch the general aspects of this emerging approach and the new insights they offer. We then provide data describing the individual cryptic morphological scaling relationships central to this approach, and investigate what these tell us about the study population. Finally, we use existing data from the literature on artificial selection applied to morphological scaling relationships to explore static allometry evolution in the context of the model, before turning our attention to areas for future work.

## The model

Briefly, the model (Dreyer et al. 2016; Shingleton 2019) employs known developmental mechanisms that regulate trait and body growth to generate a latent morphological scaling relationship for each genotype (Fig. 1A); this linear, *individual*-level morphological scaling relationship represents the continuum of size combinations for any two traits (e.g., appendages, eyes, or other organs, some proxy of body size, etc.) potentially expressed by a single genotype across the full range of possible size induced by variation in a single environmental factor (Shingleton et al. 2007). Thus, each of these genotype-specific individual scaling relationships constitutes a linear function-valued trait (Stinchcombe et al. 2012) described by a potentially unique intercept and slope. Because individuals realize just one joint size phenotype on this genotype-specific continuum, the relationship itself is not observed; the model therefore refers to these as “individual cryptic scaling relationships.”

Allelic identity at growth-regulating loci determines the intercept and slope of the individual cryptic scaling relationship, and genetic variation among individuals at these loci generates a distribution of the individual cryptic scaling relationships in the population; when populations differ in the genetic variation they harbor, the pattern of individual cryptic scaling relationships will vary among populations (Fig. 1B–D; see the original model for a formal, detailed presentation). When there is genetic variation in slopes, these distribution patterns can be divided into three categories based on the average point at which the individual cryptic scaling relationships converge (i.e., the mean point of intersection or MPI) relative to the range of trait sizes potentially expressed by each individual: the “speedometer”, where the MPI is near or below the smallest trait sizes (Fig. 1B); the “broomstick”, where the MPI is near or above largest trait sizes (Fig. 1C); and the “seesaw”, where the MPI is toward center of the size distribution, near the bivariate mean (Fig. 1D). While these categories are discrete in the model for the sake of convenience, in reality they exist along a continuum of possibilities that includes not only the location of the MPI but also how tightly the intersection points lie around it. Regardless of the distribution of the individual cryptic scaling relationships in a population, the individual phenotypes expressed on them are used collectively to fit the static allometry to the group (Fig. 1A). As discussed below, the pattern of the individual cryptic scaling relationships in a population can have wide-ranging, important effects on how the group-level static allometry responds to selection.

## The sources of size variation

The individual cryptic scaling relationship captures size covariation between traits across the full range of possible size for a single genotype. Each genotype expresses a single phenotype that corresponds to a point on their individual scaling relationship; the location of this point is determined by the level of the environmental factor experienced during ontogeny. In practice, however, measurement error and developmental instability may place the measured phenotype slightly off the genotype-specific individual scaling relationship (Shingleton 2019).

The original model (Dreyer et al. 2016) was concerned with the impact of variation in nutrition on trait and body size. This is reasonable, as nutritional variation during growth affects final size in nearly all metazoans via conserved developmental mechanisms (Oldham and Hafen 2003; Nijhout et al. 2014; Gokhale and Shingleton 2015). However, other environmental factors can also generate variation in size (e.g., temperature or oxygen level, Atkinson 1994; Frazier et al. 2001; Harrison et al. 2010; Harrison et al. 2015), and may do so in a trait-specific manner. For example, in *Drosophila*, wing size is more sensitive to temperature than it is to nutrition, relative to the rest of the body, whereas thorax size is similarly sensitive to both nutrition and temperature. Consequently, the wing–thorax individual scaling relationships are steeper when size varies in response to temperature than when size varies in response to nutrition (Shingleton et al. 2009). Further, size variation need not be due solely to environmental factors. Genetic variation in the level of systemic size regulators (e.g., growth hormones) also generates size covariation among traits (Shingleton et al. 2007), and may produce a genetic scaling relationship that is quite different from an environmental scaling relationship for the same trait pairs (Dreyer and Shingleton 2011). Thus, environmental effects on trait size, body size, and size covariation can be complex and may vary in trait- and environment-specific ways.

Trait size is not simply a developmental consequence of body size (see Gokhale and Shingleton [2015] for review). This means that scaling relationships are not equivalent to a single reaction norm where the environment is body size that determines trait size. Rather, bivariate morphological scaling relationships are the combination of *two* reaction norms, one for trait size and one for body size (Shingleton et al. 2007). Describing a scaling relationship as a single reaction norm obfuscates the scaling relationship itself with the developmental mechanisms that regulate or integrate trait and body growth to generate their individual reaction norms, confounds different types of scaling relationships (e.g., environmental versus genetic; Shingleton et al. 2007), and risks mischaracterizing the developmental and evolutionary relationship between trait and body size. To avoid implying a hierarchy of growth regulation, where body size determines trait size, below we refer to morphological allometries where the size of one trait (*x*) is plotted against the size of a second trait (*y*); *x* usually refers to some index of body size; however, it could be the size of any morphological trait.

Whether the focus is environmentally or genetically determined scaling relationships, the source of size variation matters (Shingleton et al. 2007, 2009). Ultimately, the impact of different sources of size variation on the expression and evolution of scaling will depend on the genotypic variation that resides at growth-regulating loci, the level of variation exhibited by relevant environmental factors in nature, and the degree to which these environmental factors influence phenotype expression.

## Evolutionary insight from the model

The individual cryptic scaling relationships formalize how genotype by environment interactions influence the size of traits, and how size covaries between traits across the range of possible trait sizes. Under the Dreyer (2016) model, these interactions reflect specific functional/mechanistic relationships, and genetic variation underlying these relationships structures the distribution of individual scaling relationships within populations. *More than just a static picture of observed variation, however, these distributions may account for the potentially predictable impact that selection can have on patterns of this variation, and how this affects expression and evolution of the population-level static allometry.* Evolutionary simulations using this modeling framework reveal three broad insights regarding how population-level static allometries are expected to evolve (Dreyer et al. 2016). First, and perhaps most importantly, they show that the pattern of the individual cryptic scaling relationships in a population (Fig. 1) can affect greatly the response of the population-level static allometry parameters to selection. This effect can be so strong that phenotypically indistinguishable populations—i.e., those that have the same trait size distributions, trait size covariances, and population-level static allometries—that differ in their underlying distributions of individual cryptic scaling relationships can evolve differently in response to the same pattern of selection; a single pattern of selection can produce steep or shallow slopes (i.e., hyper- or hypoallometry) or generate no response at all, depending on the distribution of the individual scaling relationships in the population. Collectively, such effects mean that predicting or interpreting the response of a population-level static allometry to selection may require knowledge of the distribution of the underlying individual cryptic scaling relationships.

Second, the model reveals that evolution of the static allometry slope depends on the mode and target of selection in somewhat surprising ways. Most notably, selection on the ratio of trait:trait size is thought to shift the intercept but not the slope (e.g., Wilkinson 1993). Yet this pattern of selection (called proportional selection in the model) can result in rapid evolution of hyper- or hypoallometry under some distributions of individual cryptic scaling relationships. In contrast, most models of scaling relationship evolution posit that the static allometry slope evolves in response to complex patterns of selection (e.g., Zeh and Zeh 1988; Green 1992; Petrie 1992; Bonduriansky and Day 2003; Kodric-Brown et al. 2006; Emlen et al. 2012; Biernaskie et al. 2014; Fromhage and Kokko 2014). The Dreyer et al. (2016) model demonstrates that such correlational selection will alter the slope under only a subset of conditions. Interestingly, the model indicates that directional univariate selection on absolute trait size can indirectly cause swift evolution of the scaling relationship slope, indicating that complex patterns of multivariate selection or combinations of different selection patterns may not be the only—or even the dominant—mechanism underlying slope evolution (see also Tobler and Nijhout 2010). These findings may inform studies that seek to determine the origin of trait exaggeration and the sometimes complicated fitness functions posited to produce steep scaling relationships under sexual selection (e.g., Zeh and Zeh 1988; Green 1992; Petrie 1992; Kodric-Brown et al. 2006; Emlen et al. 2012; Biernaskie et al. 2014).

Third and finally, the efficacy of, and response to, selection may be affected by the statistical method used to fit the population-level static allometry. Where such variation in the response to selection exists, using an ordinary least squares (OLS) Model I regression line as a reference to identify individuals for selection tends to result in a weaker evolutionary response than does using major axis (MA) Model II regression techniques (e.g., see Fig. 4 in Dreyer et al. 2016). This may be because, when compared with other line-fitting approaches, MA regression better captures the relationship between observed trait values and the underlying distribution of possible phenotypes from the individual cryptic scaling relationship in some populations (see Shingleton 2019). Regardless of the cause, this finding may add a new dimension to ongoing debates regarding appropriate regression methodologies for fitting static allometries (e.g., Frankino et al. 2009; Houle et al. 2011; Egset et al. 2012; Hansen and Bartoszek 2012; Bolstad et al. 2015; Stillwell et al. 2016; Shingleton 2019).

In sum, by taking an approach that focuses on individual-level variation in the mechanisms regulating trait growth, this modeling framework (Dreyer et al. 2016; Shingleton 2019) offers new insights into the evolution of morphological scaling. It reveals that the response to selection of the population-level static allometry slope depends on the frequency of loci that generate pattern in the distribution of individual cryptic scaling relationships in a population. Moreover, the evolutionary trajectory and speed of any response is not only determined by how the individual relationships are distributed, but also by the target of selection, and the method used to fit the population-level static allometry. Below we use isogenic *Drosophila melanogaster* lineages to describe the pattern of individual cryptic wing-body size scaling relationships for a population of flies.

## Individual cryptic scaling relationships in *Drosophila*

To quantify individual cryptic scaling relationships for several dozen *D. melanogaster* genotypes, we manipulated access to nutrition for larvae from isogenic lineages and used the resulting individuals to estimate the sex-specific cryptic scaling relationship across the full range of size for each genotype.

### System: focal traits and isogenic *Drosophila* lineages

The wing size–body size scaling relationship in *D. melanogaster* is a good model for study because it is relatively straightforward to estimate (Stillwell et al. 2011) and is ecologically relevant. The likely functional importance of wing size, body size, and relative wing size (Bastock and Manning 1955; Ewing 1961, 1964; Bennet-Clark and Ewing 1968; Dudley 2002; De Jong and Bochdanovits 2003; Frankino et al. 2009; Menezes et al. 2013; Lane et al. 2014), and geographic variation, thermally-induced plasticity, and long-term evolutionary trends in how these traits scale with one another (e.g., Azevedo et al. 1998; Gilchrist et al. 2001, 2004; Gilchrist and Huey 2004; Frazier et al. 2008; Liefting et al. 2009; Lane et al. 2014; Bolstad et al. 2015) suggest that the intercept and slope of this scaling relationship may be subject to strong and perhaps predictably variable selection.

We used flies from the Drosophila Genetic Reference Panel (DGRP), a collection of *D. melanogaster* lineages that were established from flies collected at a farmer’s market in Raleigh, NC, USA, and propagated through over 20 generations of full-sib mating to create ca. 200 isogenic lineages (Mackay et al. 2012). Below, we use “lineage” to refer to a specific isogenic line (i.e., a genotype) from the DGRP. We use “population” in reference to a cohort of genetically identical individuals from a DGRP lineage. Thus, multiple populations may be used to estimate the individual cryptic scaling relationship parameters for a given genotype.

### General methods

Egg collection, larval rearing, larval diet, application of the starvation treatment, and phenotyping followed our standard lab protocols (Stillwell et al. 2011, 2016; Myers and Frankino 2012). To generate populations for estimation of the individual cryptic scaling relationship parameters for genotypes in the DGRP, we collected eggs over 12–20 h from each lineage, transferred them in lots of 50 into 7ml of fly food, and reared larvae by genotype at 22°C until application of the starvation treatment. Larvae were removed from food at precisely timed developmental stages and starved for either 0–24 h (referred as Day 0 flies) or 48–62 h (Day 2 flies) before pupation to ensure expression of the full range of wing and body and wing size for each lineage. Pupae were isolated into individual 2.5 ml epitubes that had been punctured with a 20-gauge needle for gas exchange. The resultant adults fell into two size classes where the minimally starved individuals were ∼75% larger than the individuals subject to longer starvation. The right wing and pupal case of each fly were imaged and used to estimate wing and body size, and sex-specific body (pupal)–wing size scaling relationships were fit to each lineage using Model I and Model II linear regression.

### Experimental design and statistical analysis

We used an incomplete blocking scheme; flies from each lineage were reared in populations divided among six experimental blocks, and populations from some lineages were replicated among blocks. At a minimum, each block contained populations from 12 lineages; populations from these lineages are included in all blocks.

All analyses were conducted in *R*. The scripts for the analyses, as well as the data analyzed, are available from Dryad. All size data used to fit the individual cryptic scaling relationships for each lineage were log–log transformed. Some theoretical studies suggest that Model II linear regression best captures the developmental mechanisms that generate morphological scaling between body parts in *Drosophila* (Shingleton 2019) and so, where possible, the scaling relationship between wing and pupal size was fit using this approach (*R* package: *smatr*). However, for completeness, and when testing more complex statistical models (e.g., where lineage was treated as a random factor), we fit the relationship using Model I linear regression, using maximum likelihood (*R* package: *lme4*), and Bayesian methods (*R* package: *MCMCglmm*). We excluded lineages if six or fewer individuals of each sex were measured; other cutoffs for the number of observations within lineages (e.g., 12 or 18 per sex) had no qualitative effect on our results.

## Analysis and results

### There is genetic variation in individual scaling relationships

**Fig. 2:**
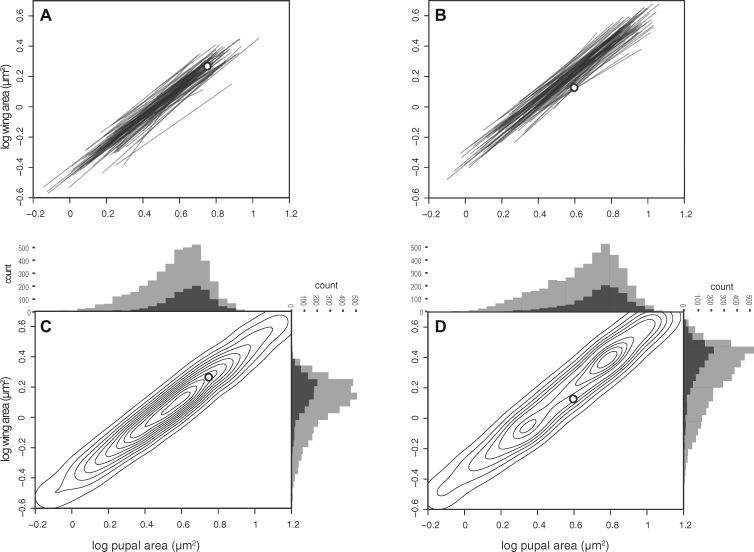
Individual cryptic scaling relationships, MPIs, and size distributions for isogenic fly lineages. The individual wing- and pupal-size cryptic scaling relationships for 85 male (**A**) and 87 female (**B**) isogenic lineages of *Drosophila melanogaster*, fit using major axis regression. The white marker indicates the mean point of intercept (MPI) among all individual cryptic scaling relationships for that sex. (**C**, **D**) Density plots (contours) for the intercepts of all pairwise combinations of individual scaling relationships for (C) males and (D) females. The histograms show the distribution of wing and pupal sizes among low-starvation flies (d0; dark gray) and all (light gray) flies. The length of the individual scaling relationships (A and B) shows the range of observed trait sizes. The individual scaling relationships as plotted do not, therefore, allow accurate visual categoraization of their pattern.

**Fig. 3:**
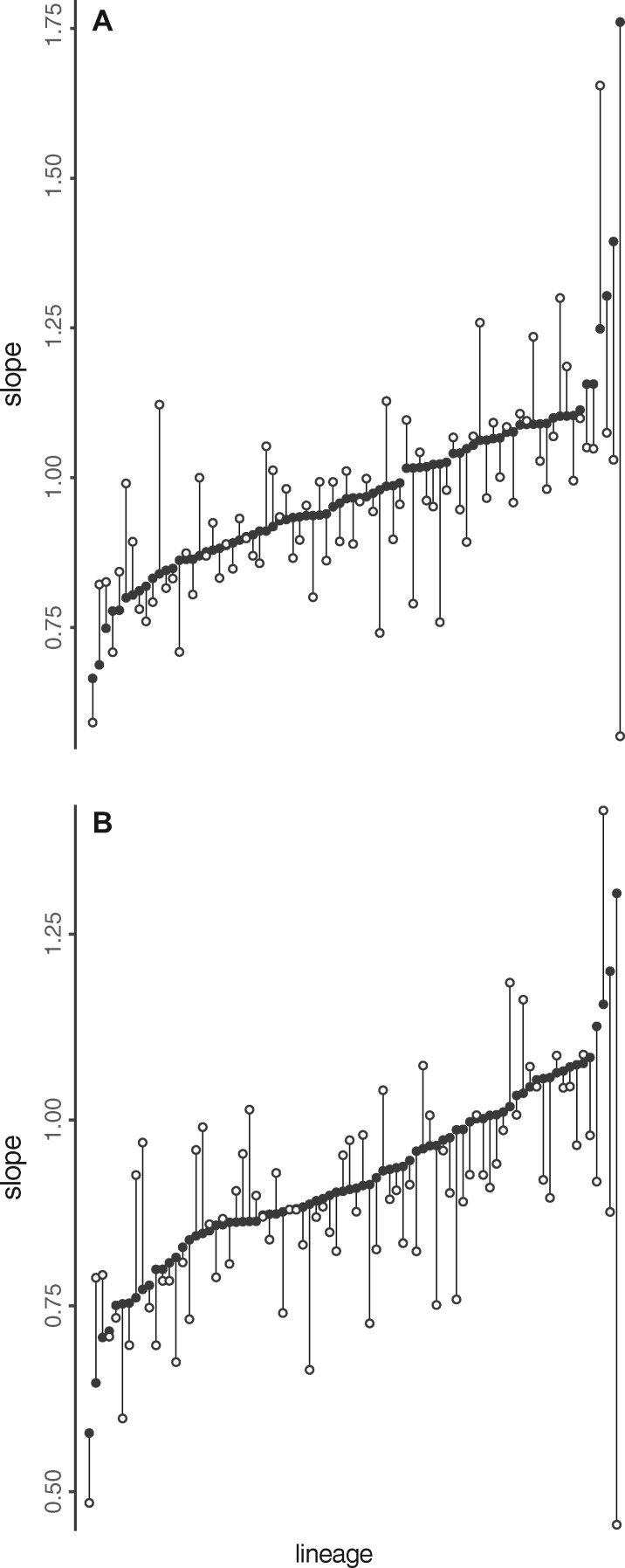
The relationship between the slope of the individual cryptic scaling relationship for males and females of the same genotype from the DGRP lineages. Each lineage is represented by the slope for females (filled circle) and males (open circles) connected by a line. Lineage slopes are sorted by value for females under (**A**) MA Type II regression and this lineage order is retained for the (**B**) OLS Type I regression slopes.

We analyzed wing- and body (pupal)-size data from 87 of the DGRP lineages. The scaling relationship between wing and pupal size differs between sexes (Table 1), and consequently we analyzed males and females separately. In both sexes, the individual cryptic scaling relationship between wing and body size varies significantly among lineages when fit using a MA Model II regression (treating lineage as a fixed factor; sma test: Wing·=·Pupa·Lineage, male: likelihood ratio*·*=*·*257.0, DF*·*=*·*80, *P* < 0.0001; Female: likelihood ratio*·*=*·*257.4, DF*·*=*·*82, *P* < 0.0001; Fig. 2) or linear mixed-model regression (treating lineage and block as a random factor, analogous to a Model I regression but with shrinkage toward the global mean slope and intercept across lineages; Table 2; figures for this analysis are presented in material on Dryad). Male and female individual cryptic scaling relationship MA Model II slopes exhibited a positive relationship among lineages (MA regression: female MA slope*·*=*·*male MA slope, slope*·*=*·*0.95, *R*^2^*·*=*·*0.1322, *P* < 0.001), and this trend held when OLS Model I slopes were analyzed similarly (OLS regression: female OLS slope*·*=*·*male OLS slope, *R*^2^*·*=*·*0.2263, *F*_(__1,78)_*·*=*·*22.82, *P* < 0.0001; Fig. 3 and Supplementary Figs. S1, S2).

**Table 1:** Effect of pupal case size and sex on wing size

**Independent** ^a^	SS	MS	DF^NUM^	DF^Den^	*F*	*P*
Sex	1.9333	1.933	1	7774.2	508.13	<0.0001
Pupa	29.0883	29.0883	1	68.1	7645.50	<0.0001
Sex: pupa	0.0528	0.0528	1	7776.9	13.87	0.0002

a Effect based on the linear mixed model *W_ijkl_*=*S_i_*+*P_j_*+*S_i_·P_j_*+*B_k_*+*P_i_·L_l_*+*ɛ_ijkl_*, where *W* is the wing size, *S* is the sex, *P* is the pupal case size, *B* is the block (random effect), *L* is the lineage (random effect), and *ɛ* is the normally-distributed error. Model was fit using *lme4* package in *R*.

**Table 2:** Effect of including random variation in slope on the fit of the relationship between wing and pupal case size in males and females

Sex	**Model** ^a^	**DF** ^b^	**AIC** ^c^	**BIC** ^c^	**Likelihood ratio** ^c^	***P*** ^d^	**Variance of slope among lineages** ^e^
Male	Model 1: *W_jkl_*=*P_j_*+*B_k_+Pj·L_l_*	7	−10,068.47	−10,024.90	68.63	0.0010	0.0075
	Model 2: *W_jkl_*=*P_j_*+*B_k_+L_l_*	5	−10,003.84	−9972.72			(0.0033–0.0117)
Female	Model 1: *W_jkl_*=*P_j_*+*B_k_+Pj·L_l_*	7	−10,860.59	−10,816.30	69.98	0.0010	0.0061
	Model 2: *W_jkl_*=*P_j_*+*B_k_+L_l_*	5	−10,794.61	−10,762.98			(0.0029–0.094)

a *W* is the wing size, *P* is the pupal case size, *B* is the block (random effect), *L* is the lineage (random effect). The models differ by having random slopes and intercepts (model 1) versus random intercepts (model 2) among lineages.

b Estimated degrees of freedom for each model.

c AIC, BIC, and likelihood ratio calculated using ML fit.

d *P*-value calculated by parametric bootstrapping using ML fit.

e Variance of slope among lineages calculated using Bayesian fit, with 95% confidence interval.

### The wing–body individual cryptic scaling relationships differ by sex

Conceptually, there are three categories of individual scaling relationship patterns or distributions: speedometer, seesaw, and broomstick (Fig. 1; Dreyer et al. 2016). While there is no clear way to capture the differences among these three patterns in a single statistic, one approach is to compare the location of the MPI with the bivariate mean of all individuals. We calculated the MPI for our DGRP lineages using the intersections of all pairwise combinations of the individual cryptic scaling relationships by sex using MA Model II regression (Fig. 2). We then used Hotelling’s two-sample *T*^2^ to test whether the MPI differed significantly from the bivariate size mean, comparing the distribution of all pairwise intersection points of the individual scaling relationships with the location of the bivariate mean of wing and pupal size. Our protocols were designed to ensure that phenotypes were represented well from across the full range of possible sizes for each genotype. This may, however, produce size distributions in our experimental population that are unrepresentative of those in nature or typical artificial selection experiments, both of which may be deficient in small individuals. To guard against any potential bias this could introduce, we conducted a conservative analysis by using two estimates of the bivariate mean: one based on all individuals and one based only on the minimally starved (Day 0) treatment individuals. For males, the MPI does not differ significantly from the bivariate mean of all individuals nor from the bivariate mean of minimally starved individuals (Table 3). In other words, the MPI is close to the center of the range of male trait sizes, suggesting their pattern of individual scaling relationships is seesaw-like (Fig. 2). In contrast, for females the MPI is significantly different from the bivariate mean trait size of all individuals, and closer to the origin, suggesting that their pattern of individual scaling relationships is speedometer-like (Fig. 2 and Table 3). The female MPI is also closer to the origin than the bivariate mean of minimally-starved individuals, but this difference is not significant (Table 3). The results were qualitatively the same when the individual scaling relationships were fit by OLS Model I regression, although the MPIs of either sex were not significantly different than the bivariate mean trait size, for all individuals or for minimally starved individuals (Table 2).

**Table 3:** Hotelling’s *T* comparing MPI for the wing–pupa individual scaling relationships to the bivariate mean of wing-pupa size for unstarved flies and all flies

	Model used to fit individual scaling relationships	Mean point of intersection	Bivariate mean of unstarved flies	Bivariate mean of all flies
			(0.63_pupa_, 0.15_wing_) SE: 0.004_upa_. 0.003_wing_	(0.52_pupa_, 0.04_wing_) SE: 0.003_upa_. 0.003_wing_

**Male**	MA	(0.75_pupa_, 0.26_wing_)	*T^2^* = 0.044, *F_2,7556_* =0.074,	*T^2^* = 0.956, *F_2,10208_* =0.478
	(Model II)	SE: 0.48_pupa_. 0.43_wing_	*P* = 0.963	*P* = 0.620
	OLS	(0.58_pupa_, 0.12_wing_)	*T^2^* = 0.141 *F_2,7656_*=0.071	*T^2^* = 0.595 *F_2,10208_*=0.298,
	(Model I)	SE: 0.26_pupa_. 0.25_wing_	*P* = 0.932	*P* = 0.742

			(0.74_pupa_, 0.36_wing_)	(0.61_pupa_, 0.23_wing_)
			SE: 0.004_upa_. 0.004_wing_	SE: 0.003_upa_. 0.003_wing_

**Female**	MA	(0.59_pupa_, 0.14_wing_)	*T^2^* = 2.139, *F_2,8082_* =1.070,	***T^2^* = 6.736. *F_2,10937_* =3.368**
	(Model II)	SE: 0.21_pupa_. 0.22_wing_	*P* = 0.343	***P* = 0.035**
	OLS	(0.17_pupa_, -0.16_wing_)	*T^2^* = 1.082, *F_2,8082_* =0.541	*T^2^* = 1.950, *F_2,_*_*10937*_ =0.975
	(Model I)	SE: 0.24_pupa_. 0.22_wing_	*P* = 0.582	*P* = 0.377

a All units are log(mm^2^).

b Significant difference is shown in bold.

**Fig. 4:**
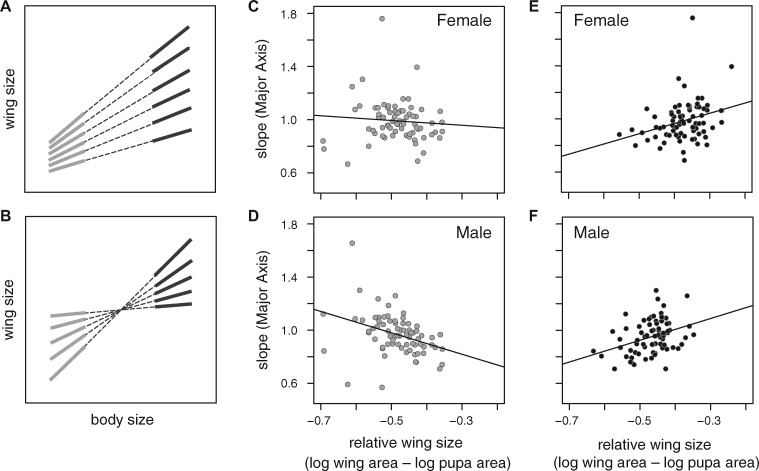
Inferring the pattern of the individual scaling relationships. (**A**) For speedometer distributions of individual cryptic scaling relationships (dashed lines) there should be a positive correlation between mean relative wing size for a lineage, based on the largest individuals (black lines), and the slope of the lineage’s individual scaling relationship, whereas this relationship should be weaker or absent among the smallest individuals (gray lines). (**B**) For seesaw distributions, the largest individuals (black lines) should exhibit a positive correlation between mean relative wing size for a lineage and the slope of the lineage’s individual scaling relationship (dashed lines), whereas the smallest individuals (gray lines) should have a negative correlation between mean relative wings size and slope. (**C**) For DGRP females, there is no significant relationship between mean relative wing size for a lineage, based on the smallest 25% of all individuals regardless of lineage, and the slope of the lineage’s individual scaling relationship (linear model: slope* *=* *relative wing size, *R*^2^* *=* *0.0066, *F*_(1,70)_* *=* *0.4664, *P *=* *0.4969), (**D**) whereas this relationship is negative for DGRP males (linear model: slope* *=* *relative wing size, *R*^2^* *=* *0.1316, *F*_(1,71)_* *=* *10.76, *P *=* *0.0016). For both females (**E**) and males (**F**), there is a significant positive relationship between mean relative wing size for a lineage, based on the largest 25% of all individuals regardless of lineage, and the slope of the lineage’s individual scaling relationship (linear model: slope* *=* *relative wing size, male: *R*^2^* *=* *0.1252, *F*_(1,74)_ =* *10.59, *P *=* *0.0017; female: *R*^2^* *=* *0.0914, *F*_(1,74)_* *=* *7.45, *P *=* *0.0079). Thus, the shading in panel A corresponds to that of the symbols in C and E, and the shading in panel B corresponds to that of the symbols in D and F.

Under the speedometer distribution of individual scaling relationships, lineages with larger mean relative wing size in the largest size classes should have steeper individual cryptic scaling relationships (i.e., the mean relative wing size of the larger size classed individuals from a lineage and the lineage’s individual scaling relationship slope should be correlated positively), whereas there should be only a weak (if any) relationship between mean relative wing size and the slope of the individual cryptic scaling relationships in the smaller size classes (Fig. 4A). In contrast, for the seesaw distribution, there should be a positive relationship between the slope of the individual scaling relationship and mean relative wing size among the largest individuals, but this relationship should be negative among the smallest individuals (Fig. 4B). We found a significant negative correlation between the mean relative wing size of a lineage—using data from the smallest 25% of all individuals, based on pupal size—and the slope of the lineage’s individual scaling relationship in males but not in females (Fig. 4C, D). In contrast, we found a significant positive correlation between the mean relative wing size of a lineage—using data from the largest 25% of all individuals based on pupal size—and the slope of that lineage’s individual scaling relationship for both females and males (Fig. 4E, F). These findings are consistent with the analyses of the sex-specific individual cryptic scaling relationship distributions described above.

With the caveat that these statistical analyses may imperfectly capture differences between groups in the pattern of individual scaling relationships, collectively our data suggest that the pattern of individual scaling relationships is speedometer-like in females and seesaw-like in males. However, closer examination reveals an interesting relationship between these sex-specific distributions. The MPI does not differ significantly between males and females (Hotelling’s two-sample *T*^2^-test, *T*^2^ = 0.496, *F*_2,__1__3__941_ = 0.248, *P *=* *0.780). This, combined with the observation that the slopes of the individual cryptic scaling relationships are correlated between sexes among lineages (Fig. 3, and Supplementary Figs. S1 and S2), suggests that males and females of the same lineage occupy different size ranges on more-or-less the same individual cryptic scaling relationships that are distributed as a seesaw (compare the size distributions, individual scaling relationships, and locations of the MPI by sex and see caption for Fig. 2). In other words, males appear on these seesaw-distributed cryptic individual scaling relationships from the smaller wing and pupal sizes to just beyond the MPI. Females, however, essentially occupy the larger pupal and trait sizes on this distribution of cryptic scaling relationships—the area where the individual cryptic scaling relationships fan outward and to the right from the MPI. Their location in the region of larger trait and body sizes on the overall seesaw distribution means that females effectively experience a speedometer distribution.

## Discussion

Many empirical and theoretical studies, some discussed above, have sought to determine how different patterns of selection might alter morphological scaling relationships to produce morphological diversity. Perhaps because the parameters of the allometric equation describe how traits scale within a biological group, and because individuals do not express a scaling relationship slope or intercept, historically this work has focused more on the population-level static allometries than on the individuals used to fit these scaling relationships. However, recent empirical studies assume particular models of relative trait growth that produce individual scaling relationships, and they assume that (genetically determined) variation underlying growth parameters may produce variation among individual scaling relationships (Egset et al. 2012; Bolstad et al. 2015). The emerging framework described here formalizes this approach to explicitly model how genetic variation among individuals in the developmental mechanisms that regulate trait growth affects the expression and evolution of morphological scaling. Under this approach, genotype determines the continuum of trait and body size covariation potentially expressed by an individual across a gradient of some environmental factor. This continuum constitutes an individual cryptic scaling relationship for that genotype, whereas the realized phenotype of the individual is a single point (set of trait values) on that continuum, determined by the environment the individual experienced (Fig. 1). Genetic variation among individuals at growth-regulating loci generates the distribution of the individual cryptic scaling relationships in the population, the pattern of which can affect strongly the response to uni- and multivariate selection.

Our analyses of the individual cryptic wing–body size scaling relationship distribution for a population of *D. melanogaster* reveal variation among genotypes in the slopes of these relationships, and a positive correlation between the slopes for males and females within lineages. For our study population, the individual cryptic scaling relationships in males seem to be distributed in a seesaw-like pattern, where their mean point of convergence is near the bivariate mean for body and wing size. Females appear to exhibit a speedometer-like distribution, where the individual cryptic scaling relationships fan outward and upward from the mean point of convergence. However, sexual size dimorphism and the correlation of slope between sexes among lineages means that females essentially occupy the larger body size–wing size portions of the same seesaw distribution occupied by the smaller sized males.

While the individual cryptic scaling relationships have not been estimated for any population subject to selection, it still may be worthwhile to review, in the context of the Dreyer et al. (2016) model, experiments that have selected to change population-level scaling relationship parameters. Below, we review in this framework some experiments that have used artificial selection to alter the parameters of morphological scaling relationships. We then turn attention toward describing the kinds of data that are needed to test the model directly.

Robertson (1962) was the first to select on body proportion directly in this context. Using *D. melanogaster*, he selected on the wing:thorax ratio—that is, wing size relative to body size—in both directions for 10 generations. Although the pooling of data between sexes and the omission of scaling relationship visualizations from the publication makes the details of the response to selection difficult to decipher, he generated populations of flies with divergent wing:thorax ratios and scaling relationship slopes. Under the Dreyer et al. (2016) model, such a response is expected when size ratios are selected from a speedometer pattern of cryptic individual scaling relationships—a distribution consistent with our observations for females from the DGRP lineages (with the caveats that we are using different measures of wing and body size than Robertson (1962), and that our population is unusual in that it was created from flies collected from a farmer’s market and then inbred). Intriguingly, Wilkinson (1993) selected on the eyestalk span:body length ratio on a natural scale to change the scaling relationship intercept in male stalk eyed flies. In addition to changing of the intercept as intended, the slopes of the selected groups diverged. However, there is no observable difference in slope between selected directions when these data are log–log transformed under MA Model II regression, and the results are equivocal under Model I regression (see analysis on Dryad https://doi.org/10.5061/dryad.f8320d5). Thus, selecting on trait size ratios can clearly alter the intercept of morphological scaling relationships, although the effect this might have on the slope is unclear, as is the role of the distribution of individual cryptic scaling relationships in determining these responses.

Most studies that have employed artificial selection to alter body proportion have used an individual’s distance in morphospace from the population-level static allometry as the basis of a selection index; individuals with extreme residuals from the group-level static allometry, sometimes weighed by their location in the size distribution along *x* or *y*, are retained to breed the next generation. This general approach has been used to change intercepts (Frankino et al. 2005, 2007; Egset et al. 2012; Bolstad et al. 2015; Booksmythe et al. 2016), slopes (Egset et al. 2012; Stillwell et al. 2016), and even the inflection point of curvilinear scaling relationships (Emlen 1996).

Three studies reported expected responses of the intercept when selection was focused on the residual distance of each individual perpendicular to the replicate-specific static allometry fit to each sex via reduced MA regression (Frankino et al. 2005, 2007; Booksmythe et al. 2016). Additionally, Kotrschal et al. (2013) observed a 9% divergence in the intercept of brain–body size allometry in guppies after just two generations. In these studies, the slopes either did not change or did so to varying degrees and direction among treatments and replicates within studies (see reanalysis of log–log transformed data on Dryad from both Frankino et al. 2005, 2007). Such inconsistent responses of the slope suggest that these shifts are not the result of an indirect response of this parameter to selection on the intercept. Two additional studies—Egest et al. (2012) and Bolstad et al. (2015)—effectively targeted the intercept of the static allometries using a more complicated index for selection that included residuals relative to the static allometry. In these two studies, the intercepts responded rapidly to selection and slope remained unchanged, demonstrating unequivocally that the intercept can evolve independently of the slope. Under the Dreyer et al. (2016) model, directional selection on the relative trait size as described here (proportional selection in the model) will not affect the slope of the scaling relationship when the distribution of individual cryptic scaling relationships is a seesaw and/or when selection is focused on individuals that are close to the MPI for the individual scaling relationships (which is close to the bivariate mean under the seesaw distribution). Interestingly, Bolstad et al. (2015) intentionally selected individuals with extreme residuals that were close to the bivariate mean. In all these studies, the consistent response of the intercept paired with the lack of any, or any consistent, response in slope suggests that the trait pairs under consideration in each may possess a seesaw distribution.

Selecting to change the population-level scaling relationship slope, and generating a response, has proven more challenging than using artificial selection to change the intercept. Two studies—Bolstad et al. (2015) and Stillwell et al. (2016)—sought to change the scaling relationship slope directly. A third study (Egset et al. 2012) also attempted to alter the scaling relationship slope, but methodological issues make interpretation of their findings less clear and so this study is not considered here in detail. Although Bolstad et al. (2015) and Stillwell et al. (2016) employed different selection procedures, both had the explicit aim of evolving the slope while keeping the mean size of both traits constant; this was accomplished by rotating the slope around the bivariate mean. In both studies, the response to selection was erratic but detectable, changing by ca. 0.5–1.0% per generation—weaker (Stillwell et al. 2016) and less consistent (Bolstad et al. 2015; Stillwell et al. 2016) on average than the response in studies that targeted the intercept. One possible explanation for this difference is that the slope of morphological scaling relationships may experience greater relative developmental or genetic constraint than does the intercept (Schwenk and Wagner 2003; Voje et al. 2014; Pélabon et al. 2014; Houle et al. 2019). This may be an unlikely explanation for at least three reasons. First, selection on relative trait size can sometimes change the slope indirectly (e.g., Robertson 1962; Weber 1990, and see Voje et al. 2014), suggesting an absence of strong constraints. Second, developmental genetic manipulations alter the slope of trait–body scaling in *D. melanogaster* (e.g., Tang et al. 2011; Shingleton and Tang 2012), suggesting slope evolution could occur among taxa through these evolutionarily conserved genetic pathways. Finally, the slopes of scaling relationships evolve among species over the longer term (e.g., Burkhardt and de la Motte 1985; Baker and Wilkinson 2001; Emlen et al. 2005; Swallow et al. 2005; McCullough et al. 2015; see reviews in Emlen and Nijhout 2000; Frazier et al. 2008; Voje and Hansen 2013; Voje et al. 2014), providing evidence for the absence of absolute constraint. On the other hand, the mechanisms that regulate scaling relationship slope appear to affect absolute trait size in tandem (Tang et al. 2011; Emlen et al. 2012; Shingleton and Tang 2012; Warren et al. 2013), and therefore may be less likely or unable to contribute to slope evolution without also affecting the bivariate mean. Consequently, a response to the selection regime used in these studies may require evolution of coordinated, condition (size)-dependent expression of different growth regulators; this level of developmental integration may constitute a constraint that would likely be difficult to overcome, and may help explain the low response to selection in these studies. However, it is important to note that the patterning mechanisms that determine intra-organ trait size (e.g., the size or location of wing veins relative to the size of the wing itself, the traits subject to selection in Bolstad et al. 2015) may be distinct from those that determine inter-organ size (see Houle et al. 2019); thus, it is difficult to draw generalities about constraint from just two studies that are focused on such developmentally distinct morphological scaling relationships.

An alternative to the constraints hypothesis is that the relative lack of response resulted from the use of a selection regime that is ineffective when applied to the distribution of individual cryptic scaling relationships present in these populations. The model predicts that correlational selection will have only moderate efficacy when applied to a seesaw distribution of individual cryptic scaling relationships, and will generate an inconsistent response when applied to populations possessing a speedometer or broomstick distribution, depending on the direction of selection and method used to fit the scaling relationship (Dreyer et al. 2016). Our data suggest that the distribution of wing–body size individual cryptic scaling relationships in the DGRP population is seesaw-like for males and speedometer-like and females (Fig. 2A, B); if this distribution holds across populations, it may explain the observed moderate response to selection on the slope of this relationship (Stillwell et al. 2016). While we do not know the pattern of individual scaling relationships for the traits from the population subject to selection in the Bolstad et al. (2015) study (but see Houle et al. 2019), similar distribution effects could also explain their results.

**Fig. 5:**
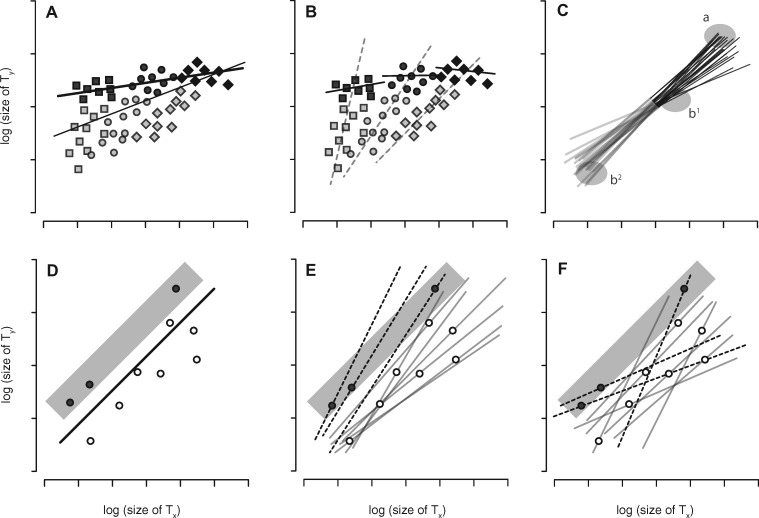
Consequences of environmental effects on phenotypic variation. (**A**) Under benign environmental conditions, individuals from three genotypes (differently shaped symbols) will approach their genetically determined maximal size (bold symbols), which is larger than the size expressed in less benign environments (light symbols). A population-level static allometry fit only to individuals from the benign environment (heavy line) may therefore differ substantially from that fit to a population of individuals reared across all environments (light line). (**B**) Individual cryptic scaling relationships fit to these genotypes may also differ depending on whether they are fit to individuals reared in a single benign environment (solid lines), or to individuals reared across all environments (dashed lines). (**C**) The range of trait sizes expressed in a population will determine the realized pattern of individual cryptic scaling relationships and thereby affect how individuals are exposed to selection as well as the response to selection itself. Here, the pattern of individual scaling relationships is a seesaw across the full range of sizes (lines), but a speedometer for populations in benign environments where all individuals are large (dark portion of lines). Selecting to rotate the slope of population scaling relationship around the bivariate mean (e.g., as in Stillwell et al. 2016) would select individuals from regions a and b^1^ in the benign environment, but would select individuals from regions a and b^2^ for populations expressing the full range of trait sizes. Individuals in regions b^1^ and b^2^ have very different individual scaling relationships, thus, the response to the same form of selection will likely differ under these ranges of environmentally induced phenotypic variation. (**D**) A population of individuals (circles) and their population-level static allometry (line). Individuals in the shaded area would be selected under proportional selection to increase the intercept. (**E**) This shows the same individuals and pattern of selection as in the previous panel, but here the individual cryptic scaling relationships are plotted (gray lines), distributed as a speedometer. (**F**) Again, the same individuals and pattern of selection in the previous two panels is shown, however, the pattern of individual scaling relationships here is a seesaw. Note that the selected individuals (filled circles) in both E and F would contribute differently to evolution of the intercept because they differ in the pattern of their individual cryptic scaling relationships (dashed lines).

More generally, the pattern of underlying individual scaling relationships, and the range of trait sizes expressed along those relationships, may strongly influence the response to selection. In nearly all the artificial selection experiments reviewed above, individuals were reared under conditions designed to minimize stress and thereby maximize survival. This makes sense in the context of artificial selection experiments where every individual is valuable, where inbreeding can reduce lineage fitness during the course of the experiment (e.g., Emlen 1996), and where the value of the experimental lineages increases with each generation. But rearing individuals under uniform or low-stress conditions is unlikely to produce the full range of possible trait and body sizes; this has at least four implications for interpreting the results of artificial selection experiments. First, rearing individuals under benign conditions means that most will grow to be near their genetically-determined maximal size; a scaling relationship fit only to individuals at the largest portion of the potential size distribution may differ from, or even misrepresent, the static allometry fit across the full range of body size (Fig. 5A). It follows that fitting a scaling relationship to only the largest individuals could also adversely affect estimations of the pattern of individual cryptic scaling relationships, the location of the MPI, etc. (Fig. 5B). Second and related, without environmental manipulation, related individuals will experience the same environmental conditions, and may therefore tend to cluster together in morphospace. Consequently, some sibships may be over- or under-represented in the selected group—not only because of their genotype, but also because the common developmental environment produces similar phenotypes among related individuals (Fig. 5A, B). In the context of artificial selection experiments, this could lead to inbreeding or otherwise alter the response to selection. Third, under conditions where there is little environmentally-induced variation in body size, selection is likely to focus more on the genetic static allometry than on the environmental static allometry (Shingleton et al. 2007) of the group. Selection on genetic static allometries may target different developmental mechanisms than selection applied to environmental static allometries, and this in turn could affect the response to selection. Finally, the range of expressed trait sizes relative to the MPI can determine the pattern of individual scaling relationships that are exposed to selection. This means that artificial selection experiments that employ only a single developmental environment may draw individuals from different subsets of the individual cryptic scaling relationships when compared with designs that include environmental manipulations that affect size (Dreyer et al. 2016). For example, the pattern of individual scaling relationships across the full range of potential trait sizes may be a seesaw, but a population of well-fed (i.e., large) individuals may occupy only the speedometer-like fan of this distribution (Fig. 5C). Such environmentally-induced variation in size distributions would expose different portions of the pattern of individual scaling relationships among populations, which in turn could produce different responses to the same pattern of selection across experiments.

The potential impact of the rearing environment on the response to selection merits further consideration. In their experiment designed to rotate the slope about the bivariate mean, Stillwell et al. (2016) applied a developmentally-timed starvation treatment to split cohorts to ensure that each selected lineage expressed the full range of wing and body size each generation, and to make certain that progeny from each cross were represented throughout the size distribution. In contrast, Bolstad et al. (2015) observed a response to selection on the slope only for populations reared on full diets; no response to selection was detected when they applied starvation treatments to their populations. This difference in response between the Bolstad et al. (2015) starved- and fully-fed companion experiments is challenging to reconcile, but it supports the hypothesis that changing the range of trait sizes expressed in the population may alter the portion of the individual cryptic scaling relationships exposed to selection. For example, if the distribution of individual cryptic scaling relationships in Bolstad et al. (2015) follows the broomstick pattern, then starved individuals will exhibit smaller trait sizes and higher variance in relative trait size, relative to fully-fed individuals, which will tend to be larger and express lower variation in relative trait size (Fig. 1C); the pattern of observed trait means, variances, and covariances will be affected differently across developmental environments under other distributions of individual scaling relationships. Regardless of the distribution of individual scaling relationships in Bolstad et al. (2015) study population, their observations emphasize the importance of considering the size range of traits in the population subject to selection, the effect that these distributions have on which genotypes—that is which individual cryptic scaling relationships—are being selected, and how these might influence evolutionary outcomes and the interpretation of results (Fig. 5C).

By definition, genotype by environment interactions mean that the phenotype expressed by each individual represents a single realization of all the possible trait values for that genotype across some environmental gradient. In most of the artificial selection studies reviewed above, populations were subject to mass selection to change the population-level static allometry based on the observed phenotype of a single individual for each genotype. Investigators selected these individuals based on their location in morphospace and the patterns of (co)variance exhibited by the traits in the population. This means that implicitly or explicitly, investigators made assumptions regarding how individuals might contribute to the response to selection. In effect, assumptions were made regarding the selected individual’s cryptic scaling relationship, and by extension, how the individual cryptic scaling relationships are distributed in the population. Our data suggest that the pattern of individual scaling relationships may vary among groups in ways that the Dreyer et al. (2016) model predicts will affect the response of the population-level static allometry to selection (Fig. 5).

In some sense, these issues surrounding the response to selection are reflected in family-based versus mass-selection artificial selection approaches. Recently, Stewart and Rice (2018) described a large, mass-selection design to alter sexual size dimorphism. Sexual size dimorphism, like morphological scaling relationships, cannot be estimated for individuals. In their experiment, Stewart and Rice (2018) did not observe any evolutionary change in the pattern of sexual size dimorphism until after 100 generations of individual-based selection. In contrast, a family-based artificial selection experiment produced rapid divergence in sexual size dimorphism in just 15 generations (Bird and Schaffer 1972). In the latter case, the “cryptic” phenotype was the sexual size dimorphism function for each genotype (family), and using this as the basis of selection enabled much more rapid evolution of the trait relative to selection based on individual-level phenotypes. In the context of scaling relationship evolution, this suggests that making predictions regarding the response to selection based on the distribution of individual cryptic scaling relationships may be more accurate than using the patterns of trait size (co)variance. Consequently, selecting individuals based on the underlying function of their individual cryptic scaling relationships may prove more profitable than selecting based their location in morphospace.

### Areas for future work

Artificial selection has proven a useful tool for exploring how morphological scaling relationships evolve. An emerging, formal focus on variation among individuals in the mechanisms that regulate trait and body growth have offered new insights regarding the expression and evolution of morphological scaling. In this context, below we outline pressing areas that need to be addressed to move forward our understanding of how morphological scaling relationships evolve.

#### I: Describe the distributions of individual cryptic scaling relationships

In developing this study, we had hoped to use existing data to describe the distribution of individual cryptic scaling relationships for a few systems where selection had been used to alter morphological scaling relationship parameters, and to employ these to determine if the distributions explained aspects of the response to selection. We were unable to do so, because those data simply do not exist. For many study systems, full sib groups can be used as proxy for genotype to estimate individual (sibship) cryptic scaling relationships in a manner similar to how split sibships can be used to estimate genetic variation in plastic responses (e.g., Newman 1988; Blanckenhorn 1998). However, the requirements for such datasets to be useful are difficult to meet. To be most effective, such studies require enough observations within sib groups to ensure a robust estimation of the individual (sibship) cryptic scaling relationships. Moreover, sib groups should be split among diets or other relevant developmental environments to produce the full range of size variation for each trait, as this will enable categorization of the individual scaling relationship distribution (i.e., seesaw, broomstick, or speedometer). Finally, a large enough number of sib groups is needed to provide sufficient power to detect variation among cryptic scaling relationship intercepts and slopes, and to allow estimation of the mean intersection point among individual scaling relationships.

Existing data that might have been used to complement studies of selection on scaling relationship parameters lacked sufficient within-sibship sample sizes or did not include environmental treatments to produce a range of trait sizes. For example, Vega-Trejo et al. (2018) contains the kinds of data that could be used to generate individual cryptic scaling relationships for the population subject to selection on relative gonopodium length in mosquitofish (Booksmythe et al. 2016); while the numbers of sibships in Vega-Trejo et al. (2018) study is impressive, and sib groups were split between diets that generated variation in adult size, there are too few observations within sibships to allow robust fitting of their individual (sibship) cryptic scaling relationships. Unfortunately, such limitations exist for every published and unpublished dataset we examined for the current study, primarily because the experiments that generated these data were designed to address other topics. Thus, experiments must be designed explicitly to address fundamental questions regarding the distribution of individual cryptic scaling relationships within populations, the degree to which different traits share distributions within populations, and how these patterns vary among populations, species, or other biological groups.

#### II. Formally test the model predictions

Once distributions of individual cryptic scaling relationships are known for a population, artificial selection can be applied to test specific predictions of the Dreyer et al. (2016) model. Such tests include determining how the distribution of individual cryptic scaling relationships affects scaling relationship evolution; to do this, populations that differ in their distributions for the same trait pairs can be subjected to the same pattern of selection (e.g., Fig. 5D–F). Tests also include determining how easily scaling relationship slopes evolve under patterns of univariate versus multivariate selection. For the reasons outlined above, a family-based approach (e.g., Bird and Schaffer 1972) could enable estimation of the distribution of individual (sibship) cryptic scaling relationships and a family-based selection approach may prove profitable. It may seem critical to use a split-cohort design in any such experiment, where individuals from each cross are reared under different developmental environments to ensure that progeny from all crosses are represented across the full range of trait sizes, and can therefore potentially contribute to the response selection (Fig. 5). However, consideration of environmental effects on size and how this affects the response to selection may benefit from a more nuanced approach, as outlined below.

#### III. Determine the role of environmental variation in shaping the response to selection

The distribution of size phenotypes expressed in natural populations may not span the full range of possible expression or could be skewed, and this could have strong effects on the response of the static allometry parameters to selection. Such possibilities can be explored empirically in a three-step program. First, the trait size distributions should be assessed in natural, focal populations; this will provide context for the remaining work. Second, using the methodology described above, the distribution of individual cryptic scaling relationships should be determined for these populations. Finally, artificial selection can be applied to determine if the response to the same pattern of selection holds when applied to different subsets of the full individual cryptic scaling relationships distributions. The results of Bolstad et al. (2015) suggest that the response may not be the same for populations that differ in the pattern or range of phenotypic variation they express. It is important to elucidate why this is so. The expected response of the population-level static allometry to selection will likely differ if populations express only a partial range of body and trait size, or if selection sees only a limited or biased portion of the size distribution—thereby effectively converting one distribution of the individual cryptic scaling relationships into another (e.g., turning a seesaw distribution into a speedometer; Fig. 5C).

In conclusion, by focusing attention on the patterns of cryptic individual-level variation that underlies observed population-level scaling relationships, the framework we present provides a different approach to study the expression and evolution of morphological scaling. The emerging models that use this framework suggest wide ranging, counterintuitive, and important insights. Evaluating the validity of these insights is an empirical task, and applying selection to carefully constructed artificial populations where the distribution of individual scaling relationships has been defined is likely to be a key tool in this undertaking.

## Supplementary Material

icz135_Supplementary_Data
