# Supplementary material for: Individual Cryptic Scaling Relationships and the Evolution of Animal Form
Source: Integr Comp Biol. 2019 Jul 31;59(5):1411–28. doi: 10.1093/icb/icz135 (PMC6863759; doi:10.1093/icb/icz135)
Supplement: icz135_Supplementary_Data [file icz135_supplementary_data.pdf]

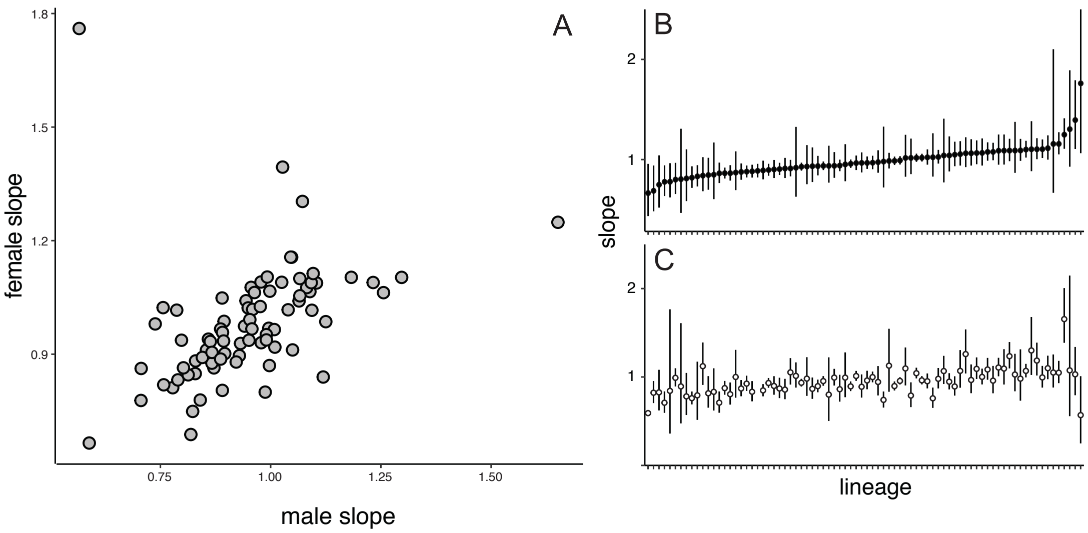

Figure S1. Relationships among lineage slopes (Type II regression). Slopes were estimated for each lineage by sex using Type II regression. (A) The relationship between male and female slopes by lineage. (B) Female and (C) male slopes with 95% confidence interval for each lineage. Slopes are sorted by size for females and male observations correspond to this order.

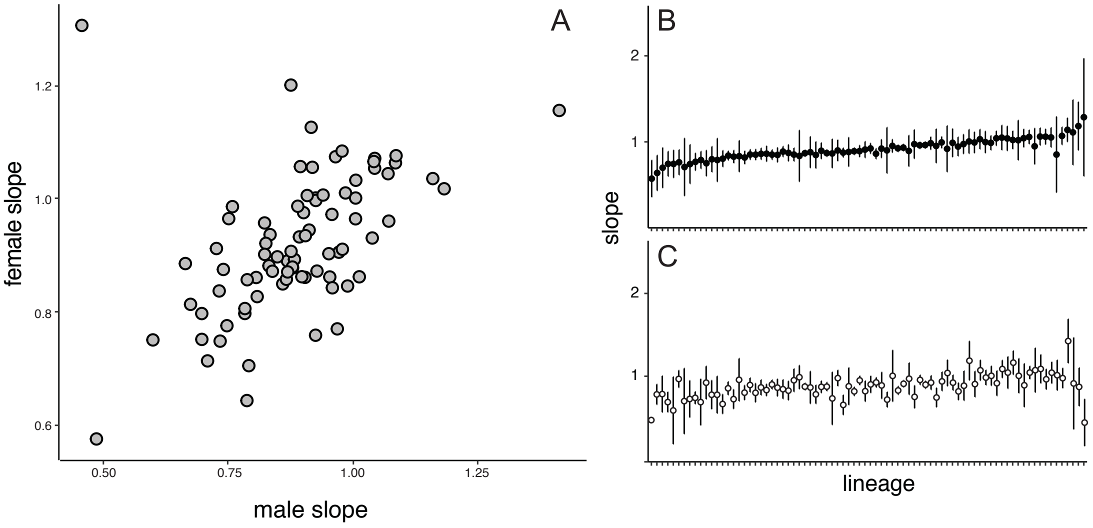

Figure S2. Relationships among lineage slopes (Type I regression). Slopes were estimated for each lineage by sex using Type I regression. (A) The relationship between male and female slopes by lineage. (B) Female and (C) male slopes with 95% confidence interval for each lineage. Sorting order for slopes from Figure S1 is retained for comparison.
